# Atomic and Electronic Structures of Co-Doped In_2_O_3_ from Experiment and Theory

**DOI:** 10.1021/acsami.4c05727

**Published:** 2024-05-29

**Authors:** Maria Voccia, Samadhan Kapse, Rocío Sayago-Carro, Natividad Gómez-Cerezo, Marcos Fernández-García, Anna Kubacka, Francesc Viñes, Francesc Illas

**Affiliations:** † Departament de Ciència de Materials i Química Física & Institut de Química Teòrica i Computacional (IQTCUB), Universitat de Barcelona, c/Martí i Franquès 1-11, 08028 Barcelona, Spain; ‡ Consejo Superior de Investigaciones Científicas, Instituto de Catálisis y Petroloquímica, Campus Cantoblanco, Madrid 28049, Spain

**Keywords:** In_2_O_3_, doping, cobalt, oxygen vacancy, bulk

## Abstract

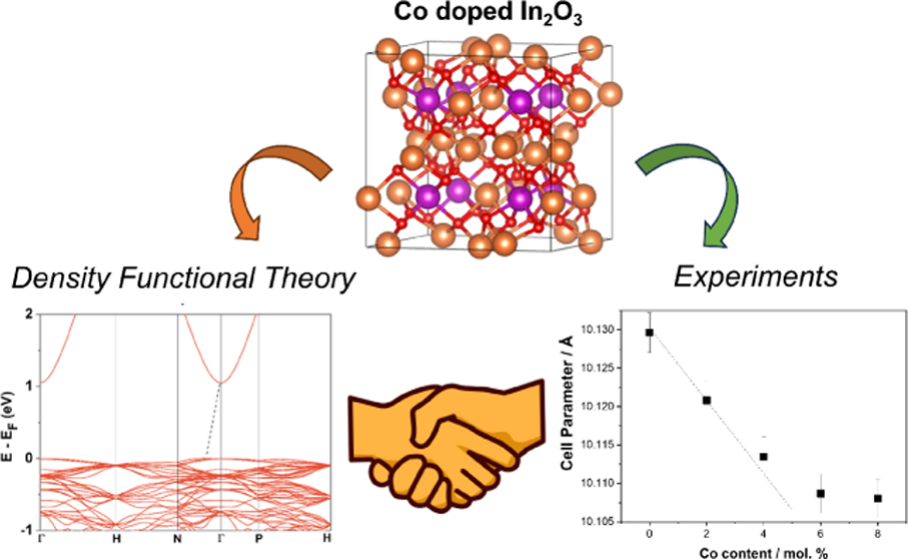

The synthesis and properties of stoichiometric, reduced, and Co-doped In_2_O_3_ are described in the light of several experimental techniques, including X-ray diffraction (XRD), X-ray photoelectron spectroscopy (XPS), ultraviolet (UV)–visible spectroscopy, porosimetry, and density functional theory (DFT) methods on appropriate models. DFT-based calculations provide an accurate prediction of the atomic and electronic structure of these systems. The computed lattice parameter is linearly correlated with the experimental result in the Co concentration ranging from 1.0 to 5.0%. For higher Co concentrations, the theoretical-experimental analysis of the results indicates that the dopant is likely to be preferentially present at surface sites. The analysis of the electronic structure supports the experimental assignment of Co^2+^ for the doped material. Experiments and theory find that the presence of Co has a limited effect on the material band gap.

## 1 Introduction

It is nowadays well established that the anthropogenic increase of CO_2_ concentration on the Earth’s atmosphere is the cause of global warming, with undesirable effects such as more frequent violent meteorological events or ocean acidification, with the concomitant harmful effects on coral reefs and marine life, to mention a few. Reducing the CO_2_ concentration in the atmosphere becomes an urgent need and requires the combination of several transversal technologies addressed at CO_2_ sequestration, or even better, at the use of CO_2_ as a C_1_ feedstock to manufacture commodity chemicals.^1^ Among the different possibilities, the so-called power to gas^2,3^ technology has gained attraction as a promising option to absorb and exploit surplus renewable energies using CO_2_ as a feedstock. However, the exceedingly large stability of the CO_2_ molecules severely hampers its chemistry, and the use of catalysts becomes unavoidable, with remarkable, yet insufficient, progress in the field.^4,5^

Among the possible catalysts for CO_2_ conversion to methanol, metal oxides exhibit some advantages as, contrarily to metals, CO_2_ adsorption can be moderately large, eventually leading to noticeable bending of the adsorbed molecule, which is a fingerprint of activation although this descriptor turns out to be insufficient.^6^ Due to its appealing selectivity and high activity toward methanol, In_2_O_3_ is among the most studied oxides for CO_2_ conversion to methanol.^7−9^ In fact, recent work on indium oxide-based catalysts for CO_2_ hydrogenation reports a 100% selectivity toward methanol at a temperature as low as 200 °C.^9,10^ Several experiments and density functional theory (DFT) investigations highlight the role of oxygen vacancies in the mechanism of CO_2_ hydrogenation to methanol on In_2_O_3_.^11−16^ Although In_2_O_3_ exhibits high selectivity toward methanol by suppressing the competitive reverse water gas shift reaction (RWGS) reaction, CO_2_ conversion and thus the yield of methanol is limited by the low activity of this material in dissociating molecular H_2_. Several promoters have been studied to improve the rate of hydrogen activation and achieve some inspiring results, including rare-earth elements^17^ and precious metals. Concerning the latter, it has been shown that the addition of small Pd nanoparticles can enhance methanol synthesis.^18^ Furthermore, small Pd clusters increase the rate of H_2_ activation, resulting in a larger amount of H adatoms at the metal–oxide interface,^19^ and similar results have been reported for Pt-promoted In_2_O_3_.^20−22^

Because of the scarcity and concomitant high cost of Pd and Pt, it is highly desirable to limit their amount by appropriate engineering as done by Frei et al.^23^ or use Earth-abundant metals such as first-row transition metals. Interestingly, a recent study has demonstrated that incorporating Co into In_2_O_3_ increases its activity and selectivity toward methanol.^24^ The strategy to use Co doping on In_2_O_3_ follows previous work where Pd is used as a dopant,^23^ and constitutes interesting alternative possibility to enhance or fine-tune the conversion of the CO_2_ hydrogenation to methanol that goes beyond the broadly used support catalysts. To date, however, the geometric and electronic structure and atomistic details of Co-doped In_2_O_3_ catalysts are lacking.

To address this issue and to provide a theoretically sound interpretation of the experimental observations, several Co–In_2_O_3_ catalysts, hereafter denoted as In_*x*_–Co, were synthesized with different In/Co ratios, while the resulting catalysts were characterized by using X-ray diffraction (XRD)–Rietveld, transmission electron microscopy (TEM), X-ray photoemission spectroscopy (XPS), and ultraviolet (UV)–visible spectroscopy. The study emphasizes the analysis of the low Co loading where maximum dispersion of cobalt would be achieved. Furthermore, we fully address the influence of Co doping on the properties of Co-doped bulk In_2_O_3_. By means of first-principles-based calculations, carried out in the framework of DFT, we analyze the structural, energetic and electronic effects of Co doping as a function of the Co dopant concentration. Ultimately, this study aims to facilitate comprehensive and systematic development in highly efficient Co-doped In_2_O_3_-based catalysts for the conversion of CO_2_ to methanol.

## 2 Experimental Procedure

A series of Co-doped In_2_O_3_ samples were prepared using a reverse microemulsion procedure. The microemulsion utilized X-100 Triton as a surfactant, *n*-hexanol as a cosurfactant, and *n*-heptane as an organic medium and water.^25^ Once the emulsion phase is formed, adequate quantities of indium and cobalt were placed in the water counterpart. Indium(III) nitrate hydrate and cobalt(II) nitrate hexahydrate (Aldrich) were used as precursors. Upon 30 min of agitation, the resulting solution was mixed with a similar microemulsion but containing tetramethylammonium hydroxide (TMH; Aldrich) in the aqueous medium. This triggers precipitation of the cations. After overnight aging, the resulting solids were cleaned with methanol, dried at room temperature, and finally calcined at 350 °C for 2 h. The solids were named In_*x*_Co, where *x* corresponds to the molar content (cation basis). The molar content was tested between 2 and 8 mol % and confirmed using atomic absorption (ICP-OES; Optima 3300DV PerkinElmer spectrometer).

XRD measurements were carried out with the help of a Bruker D8 Advance diffractometer with a Ni-filtered Cu Kα radiation (λ = 0.15406 Å) apparatus. Semiquantitative Rietveld refinement of the diffraction patterns was carried out with the FullProf program.^26^ UV–visible spectroscopy was also utilized to characterize the solids using a Varian Cary300 apparatus. The absorption coefficient of the samples was calculated using the Kubelka–Munk function.^27^ Finally, XPS measurements were done with the PHOIBOS 150 WAL hemispherical energy analyzer, an XR 50 Al-X-ray source, and a μ-FOCUS 500 X-ray monochromator. To take into account the effect of potential charging effects, the binding energies (BE) were referenced against the C 1s peak (284.6 eV). The CASA 2.3.25 software was utilized for fitting procedures. A Shirley background allowed the elimination of inelastic and other undesired contributions.^28^

## 3 Theoretical Methodology and Computational Details

The present DFT-based calculations have been carried out using the well-known Perdew–Burke–Ernzerhof (PBE) exchange-correlation functional within the generalized gradient approximation (GGA).^29^ This functional is known to duly describe the structural and energy bond features of In_2_O_3_ systems.^30^ Nevertheless, local density approximation (LDA) and GGA functionals such as PBE are known to underestimate the band gap of semiconducting oxides,^31^ and for this more accurate but computationally expensive hybrid functionals, or the more computationally economic, but semiempirical, inclusion of a Hubbard-like term as in LDA + *U* or GGA + *U* are advised. In the present work, the electronic structure properties are derived from PBE + *U* calculations,^32^ carried out in a single-point fashion, imposing a standard *U* value of 7 eV for In 4d orbitals, denoted as *U*_In_(4d), as previously reported.^16,33^ In the case of doped Co models, the same approach has been used for the electronic structure properties, and single-point calculations have been carried out with a standard value of *U* = 5 eV for the Co(3d) levels, hereafter denoted as *U*(Co_3d_). For comparison, we also report values computed without adding the *U*(Co_3d_) term.

The DFT-based calculations have been carried out using the Vienna Ab Initio Simulation Package (VASP),^34^ employing a plane-wave basis set to expand the valence electron density. Spin polarization was always taken into account, but the results consistently converged to the closed-shell solution. A kinetic energy cutoff of 415 eV has been used to select the plane-wave basis set which, according to previous studies with representative test cases with increased cutoff energies,^35,36^ which provided converged energy results with variations below 10^–4^ eV. The effect of the core electrons on the valence electron density is described by the projector-augmented wave (PAW) method.^37^ The integration on the reciprocal space was carried out using optimized grids of Monkhorst–Pack^38^ special *k*-points as described below.

**Figure 1:**
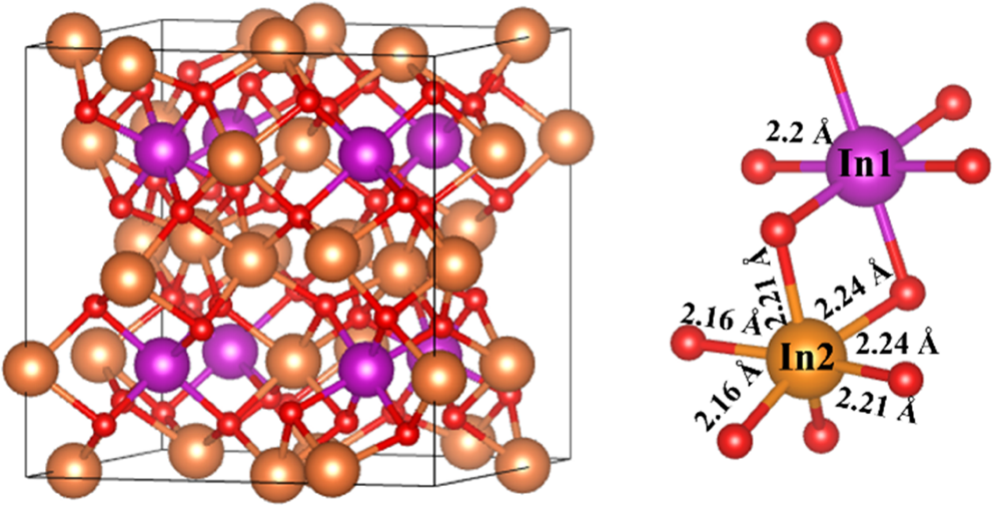
Conventional crystal structure of bcc In_2_O_3_ containing 80 atoms (left) and oxygen coordination around Indium atom (right). Orange and purple spheres represent the 32 In atoms (8 In_1_ and 24 In_2_, respectively), and red spheres represent the 48 oxygen atoms.

There are three different reported In_2_O_3_ bulk polymorphs, the most stable one being the body-centered cubic (bcc) bixbyite crystal structure (cf. Figure 1) at low temperature and ambient pressure as previously reported.^39,40^ The bixbyite conventional cubic unit cell corresponds to the *Ia*3̅ space group (#206) with a lattice parameter of 10.117 Å,^40^ and contains a total of 80 atoms, with 48 O atoms and two types of In atoms with different local environments, denoted as In_1_ and In_2_, respectively, distributed as 8 In_1_ and 24 In_2_ (cf. Figure 1). Different periodic supercells simulating different doping situations are considered. These are the (1 × 1 × 1), (2 × 1 × 1), and (2 × 2 × 1) supercells containing 80, 160, and 320 atoms, respectively. For simplicity, in the following, we will refer to these supercells as 111, 211, and 221, respectively. In all cases, all atomic and unit cell degrees of freedom were fully allowed to relax during the energy minimization procedure. Structural optimizations were considered converged when cell internal pressure was nominally below 0.01 GPa, and forces acting on atoms were below 0.01 eV·Å^–1^. To enhance the convergence of energy with respect to electron density variations, a smearing technique was employed with a tetrahedron method with Blöchl corrections with an energy window of 0.2 eV. For all structure optimization cases, an optimized 3 × 3 × 3 Monkhorst–Pack *k*-point grid was used to carry out the integration in the reciprocal space. For electronic structure calculations, an optimal 9 × 9 × 9 grid was used to acquire more accurate results.

For the doped systems, including up to *n* Co atoms substituting In, the defect formation energy per atom, *E*_f_, is defined as
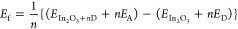
1where *E*_In_2_O_3__ is the total energy of the defect-free In_2_O_3_ bulk supercell, *E*_In_2_O_3_+*n*D_ is the total energy of the In_2_O_3_ cell containing *n* D doping atoms with *n* less In atoms, *E*_D_ is the total energy of the doping atom, here Co, taking the bulk energy per atom as reference, and *E*_A_ is the total energy of the removed In atoms, also taken from bulk In. Within this definition, negative *E*_f_ values denote energetically preferred doping situations, and vice versa. In the cases where more than one Co atom is included in the supercell, a systematic search exploring the situations with Co atoms close to or far from each other was also carried out. The effect of Co doping on In_2_O_3_ reducibility has also been assessed by computing the oxygen vacancy energy formation, *E*_O_vac__, for the stoichiometric and Co-doped supercells. In all cases, *E*_O_vac__ has been computed as in eqs 2 and 3 for the nondoped and Co-doped systems, respectively. Note also that eq 2 holds when introducing more than one vacancy to the unit cell, and the provided *E*_O_vac__ value is per vacancy formed. The same applies to the cells with more than one Co atom. Thus
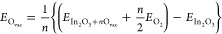
2
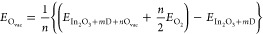
3where *E*_O_2__ is the computed energy of the O_2_ molecule in gas phase in its triplet ground state, gained *G*-point in a large box of 15 × 15 × 15 Å^3^ dimensions; *E*_In_2_O_3_+*n*O_vac__ is the energy of the supercell with *n* O vacancies; *E*_In_2_O_3_+*m*D+*n*O_vac__ is the energy of the supercell with *m* Co atoms and *n* O vacancies; and *E*_In_2_O_3_+*m*D_ is the energy of the supercell including *m* Co atoms so as to represent the concentration of interest. To gain information about the chemical bonding and how it is affected by the presence of Co atoms, we rely on net Bader charges estimated from the topological analysis of the total electron density,^41^ as well on the analysis of the density of states (DOS).

## 4 Results and Discussion

### 4.1 Experimental Section

**Figure 2:**
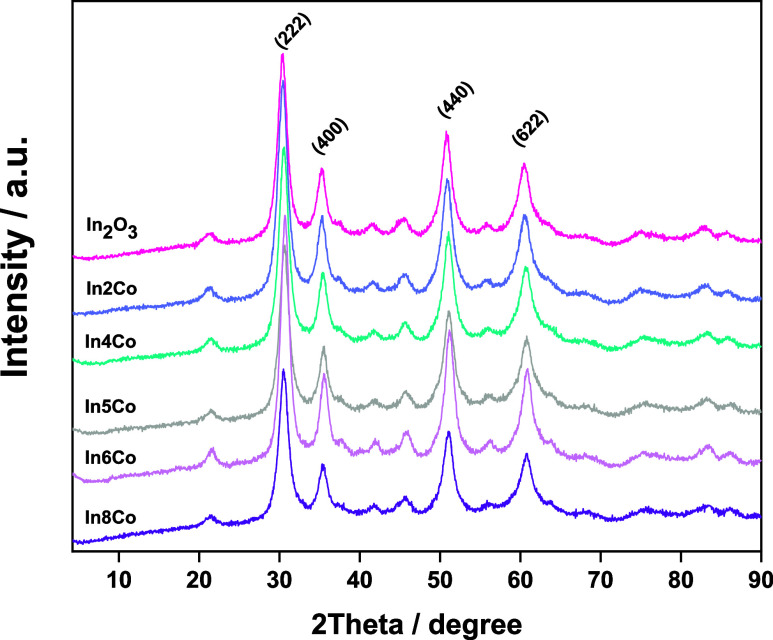
XRD patterns for the In_*x*_Co and reference samples.

**Figure 3:**
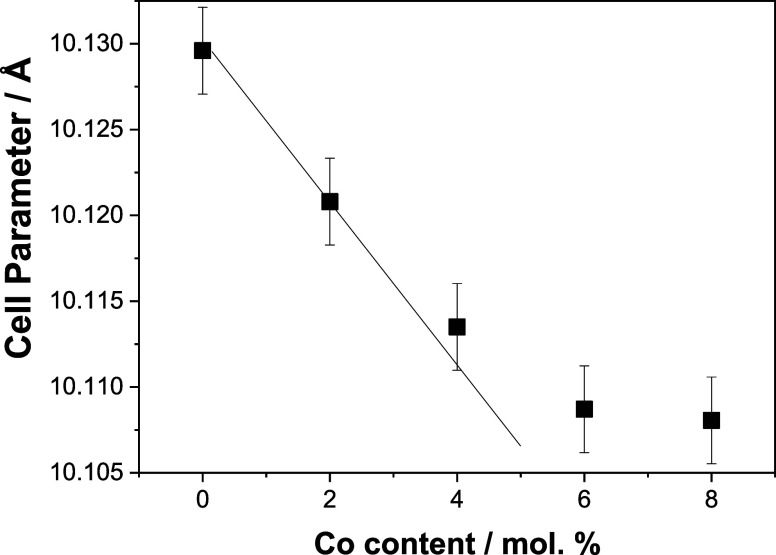
Experimental cell parameter (Å) as a function of cobalt concentration (Co/mol %) obtained by the XRD analysis.

As mentioned earlier, the XRD signal of the samples display patterns (cf. Figure 2) which can be indexed according to the body-centered cubic (bcc) bixbyite crystal structure of the indium(III) oxide, In_2_O_3_.^39,40^ Single-phase solids are thus obtained with a structure characteristic of bare indium oxide. The width of the XRD peaks clearly shows the nanostructured nature of the solids. The analysis of the patterns indicates that all samples show an essentially invariant particle size of ca. 6 nm, see Figure 2, and a cell parameter behaving as shown in Figure 3. As the main point concerning the effect of cobalt doping of the bixbyite structure, we can see that the cell parameter decreases with the cobalt content of the materials up to a level of ca. 5 mol %. After this point, a more or less stable value can be observed. The behavior of the cell parameter as a function of the cobalt content of the materials roughly agrees with previous reports concerning microsized cobalt-doped bixbyite solids.^42^ The constant values presented by the primary particle size and BET area parameters further prove the similar textural properties of the solids; see Table 1. We can thus see that the cobalt doping of the materials exerts limited effect on the structural and morphological properties of the high surface area, nanostructured solids, with main effects connected with the modification of the lattice cell parameter. This will be below interpreted with the help of theoretical tools.

**Table 1:** Particle Size (Size, in nm), Band Gap (*E*_g_, in eV), BET Area (BET, in m^2^·g^–1^), and Pore Size (Pore, in cm^3^·g^–1^) for the Different Samples in the Leftmost Column

sample	size	*E*_g_	BET	pore
In2Co	6.0	2.9	146	0.46
In4Co	6.0	2.9	150	0.43
In5Co	6.4	3.0	147	0.40
In6Co	6.2	2.9	149	0.46
In8Co	6.1	3.0	148	0.47
In_2_O_3_	6.2	2.7	146	0.46

**Figure 4:**
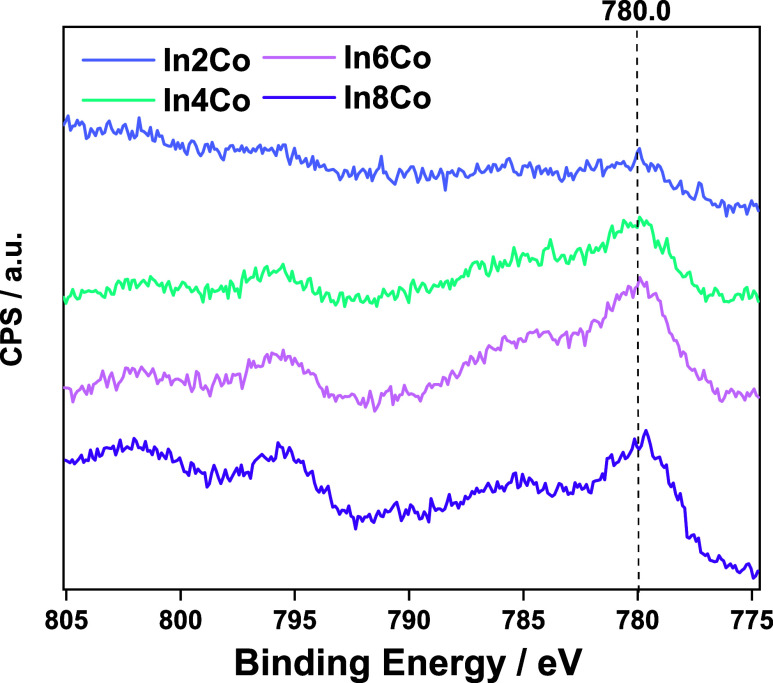
Co 2p XPS signals for the In_*x*_Co samples.

**Figure 5:**
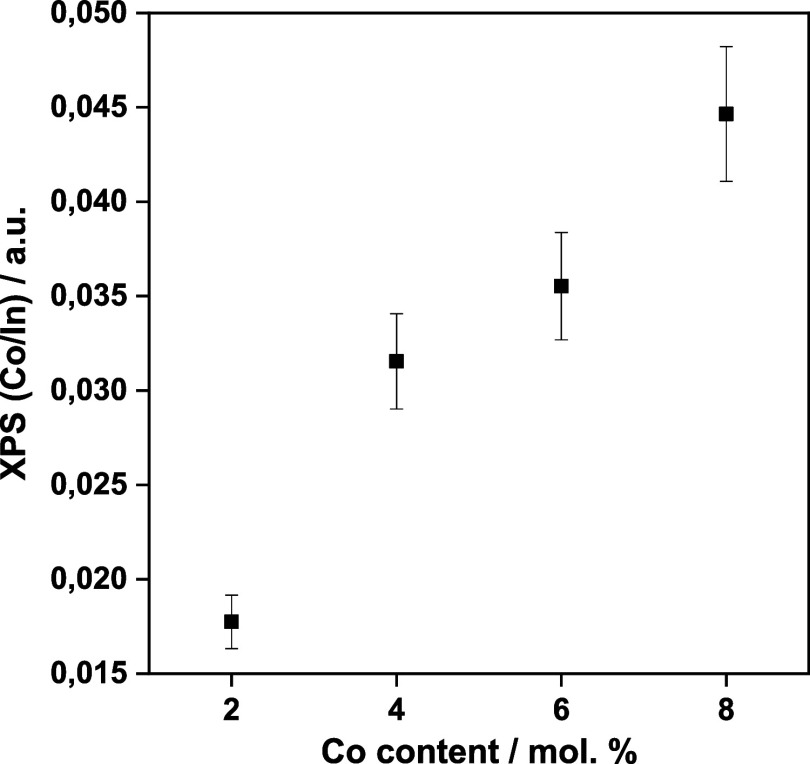
Co/In XPS ratio as a function of the cobalt content of the solids as measured by chemical analysis.

**Figure 6:**
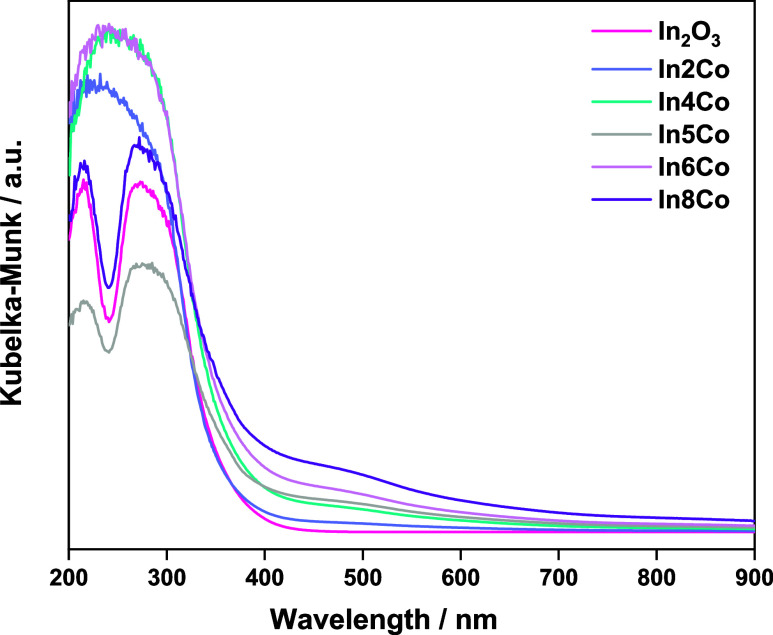
UV–visible spectra for the In_*x*_Co and reference samples.

The electronic properties of the solids were analyzed by using XPS and UV–visible spectroscopies. The Co 2p XPS signal displays a 2p_3/2_ peak with a binding energy without dependence on the Co content, see Figure 4. An invariant signal at nearly 780.0 eV is detected, which is assigned to the presence of Co(II) species in an oxidized environment. This is further corroborated by the intense shakeup satellite located at ca. 6 eV higher binding energy than the main 2p_3/2_ peak.^43,44^ The Co/In XPS ratios were studied as a function of the cobalt content of the solids, and the results are summarized in Figure 5. The plot shows a different behavior for samples below or above ca. 5 mol %. Moreover, above that point, the Co/In ratio displays values well below the expected ones (the cobalt content), indicating either a depletion of cobalt entities from the surface or, more likely, some kind of aggregation of the mentioned component. The study of the main electronic features of the solids is completed with the analysis of the band gap energy using UV–visible spectroscopy; see Figure 6. Considering nanostructured indium oxide as an indirect gap semiconductor,^45^ a value of 2.7 eV is obtained for the indium oxide reference powder. The presence of cobalt on the structure increases this value to 2.9–3.0 eV, without noticeable influence of the cobalt content of the solids. In Figure 6, we can also observe that the presence of cobalt exalts the intensity in the visible region around 400–550 nm, with a center or maximum at ca. 470 nm. This clearly points out to the presence of localized electronic states connected with defects, which will be further analyzed using theoretical tools.

### 4.2 Computational

#### 4.2.1 Properties of Undoped In_2_O_3_

**Figure 7:**
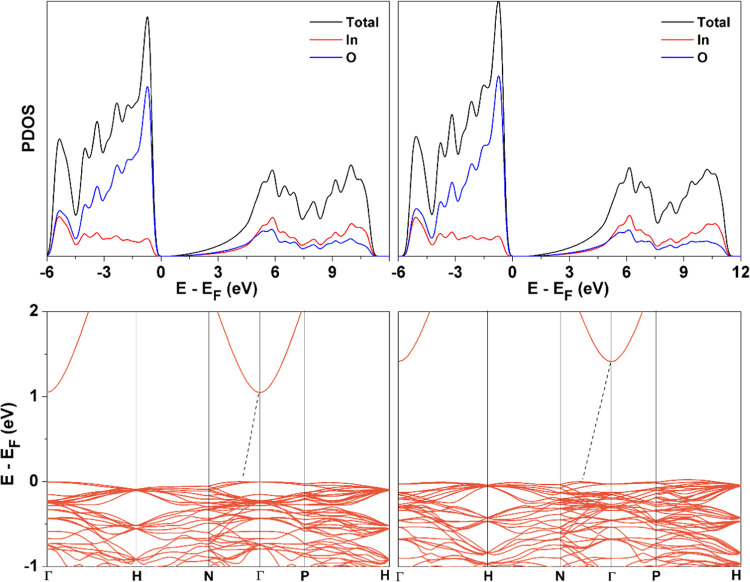
Total DOS and PDOS for bulk bcc In_2_O_3_ using PBE (top, left) and PBE + *U* (top, right) and band structure PBE (bottom, left) and PBE + *U* (bottom, right), indicating the indirect character of the band gap. All energies are referred to Fermi energy, *E*_F_, defined here as the maximum of the valence band.

We start by describing the results corresponding to the bulk structure of In_2_O_3_. The PBE optimized lattice parameter is 10.227 Å, in agreement with previous work using the same method,^30,46^ and with studies using interatomic potentials,^47^ although slightly larger than the experimental value of 10.124 Å for microsized (well-crystallized) powders,^42^ yet in good agreement with the present experimental value of 10.130 Å obtained from XRD, see Figure 2. The band structure and projected DOS (PDOS) of bcc In_2_O_3_ are shown in Figure 7. The nature of the band gap of bulk In_2_O_3_ has been a matter of debate with experiment and theory, with some contradiction related to the presence of indirect and direct bandgaps of 2.62 and 3.75 eV, respectively.^40^ However, calculations with either PBE or PBE + *U* (*U* = 7 eV) find very small differences, below 0.04 eV, between the indirect and direct gaps, with values of indirect gap around 0.93 (PBE) and 1.79 eV (PBE + *U*), as reported by Erhart et al.^48^ These calculated values are lower than those of the experiment as expected. A somehow more accurate value of 2.20 eV has been reported from calculations with the HSE06 hybrid functional by Ramzan et al.^46^ These authors also computed the imaginary part of the dielectric function and deduced indirect and direct bandgaps of 2.20 and 3.17 eV, which they claim are in line with the experiments. The present PBE and PBE + *U* values for the direct/indirect band gap are 1.06/1.05 and 1.42/1.38 eV, respectively, smaller than those predicted with the hybrid functional, as expected,^31^ and in line with previous work using a similar approach.^48^ Therefore, one can conclude that the present calculated band gap values are in qualitative agreement with the present experimental value of 2.7 eV, and in good agreement with other previous measurements.^40^ Nevertheless, one must be aware of the limitations of the PBE + *U* functional implying that the fact that calculated band gap values are in agreement with the present experimental value may be fortuitous. Also, the experimental values are affected by uncertainties.

Analysis of the DOS and band structure plots in Figure 7 shows that the DOS predicted from the PBE functional is divided into three regions. The valence band spreads over approximately 5 eV and is mainly composed of the O 2p states, with some hybridization with the In 4d states. Including the Hubbard *U* term shifts the In 4d states in the second region to lower energies by approximately 0.2 eV and increases the width of this region by ∼less than 1 eV. In this way, the effect of the *U* term is to place the In(4d) states closer to the experimental position.^49^ Moreover, a higher degree of hybridization between the In(4d) and the O(2s) states is observed in both the second and third regions. The valence band, which is dominated by the O(2p) and In(4d) states, is only weakly affected by the Hubbard correction. From the band structure in the bottom panels of Figure 7, one can readily see the indirect character of the band gap. In addition, the very small dispersion of the valence bands is consistent with a contribution of ionic bonding which is supported by Bader charges in In and O of +1.90 and −1.26, respectively. Note, however, that in spite of the ionic character, the charges are far from the formal oxidation state values of +3 and −2, respectively. Since the main goal of the present paper is to investigate the effect of Co doping in the atomic and electronic structure of the doped system, we refrain to further analyze the electronic structure of the stoichiometric material.

To end the discussion regarding the nondoped materials, we focus now on the oxygen vacancy, O_vac_, energy formation. This obviously may depend on the supercell used as it is directly related to the oxygen vacancy concentration. The lowest value of 2.51 eV corresponds to the lowest oxygen vacancy concentration of ∼0.6 atom % and slightly increases with increasing the O vacancy concentrations. Thus, for a range of O vacancies considered from 0.6 to 6 atom %, the computed O vacancy formation is at most 3 eV (cf. Table 2), and is accompanied by a very small expansion of the lattice. This is not surprising as removing one neutral O atom leads to the creation of *F* centers in In_2_O_3_,^50^ as does happen in the more ionic MgO^51^ and Al_2_O_3_^52^ oxides with electron density trapped in the generated vacancy^31^ acting as pseudoatoms.^53^ Note also that the value for O vacancy formation at a 2.08 atom % concentration as calculated with the 111 and 211 is slightly different, 2.75 and 3.07 eV, respectively, which is due to small differences in the quality of the basis set as the degree of completeness of the basis set provided by the same energy cutoff in the two supercells is different. This is also the case for the numerical integration in the reciprocal space for the two supercells, where the same *k*-point grid is employed. This small but noticeable difference provides error bars for the present estimate of this quantity. For comparison, the reported values of O vacancy formation energies of similar materials such as MgO, TiO_2_, and CeO_2_, computed also with respect to molecular oxygen as in eq 3, are 6.95, 4.05, 3.20 eV respectively.^54^ The present results indicate that even if there is no direct experimental evidence of O vacancies in the present samples, oxygen vacancies can be generated quite easily and are likely to play a role.

**Table 2:** Dimension of Supercell, Number of Vacancies and Atomic Concentration (#Ovac and Atom %), Vacancy Energy Formation, *E*_f_, in eV, and Optimized Lattice Parameter, *a*, in Å^a^

supercell	#Ovac/(atom %)	*E*_f_	*a*
111	0 (0.00)		10.227
221	1 (0.58)	2.51	10.240
211	1 (1.04)	2.91	10.243
211	2 (2.08)	3.07	10.244
111	1 (2.08)	2.75	10.243
111	2 (4.17)	2.99	10.248
111	3 (6.25)	2.98	10.238

a For situations with more than one O vacancy, the most stable situation is considered.

#### 4.2.2 Properties of Co-Doped In_2_O_3_

**Figure 8:**
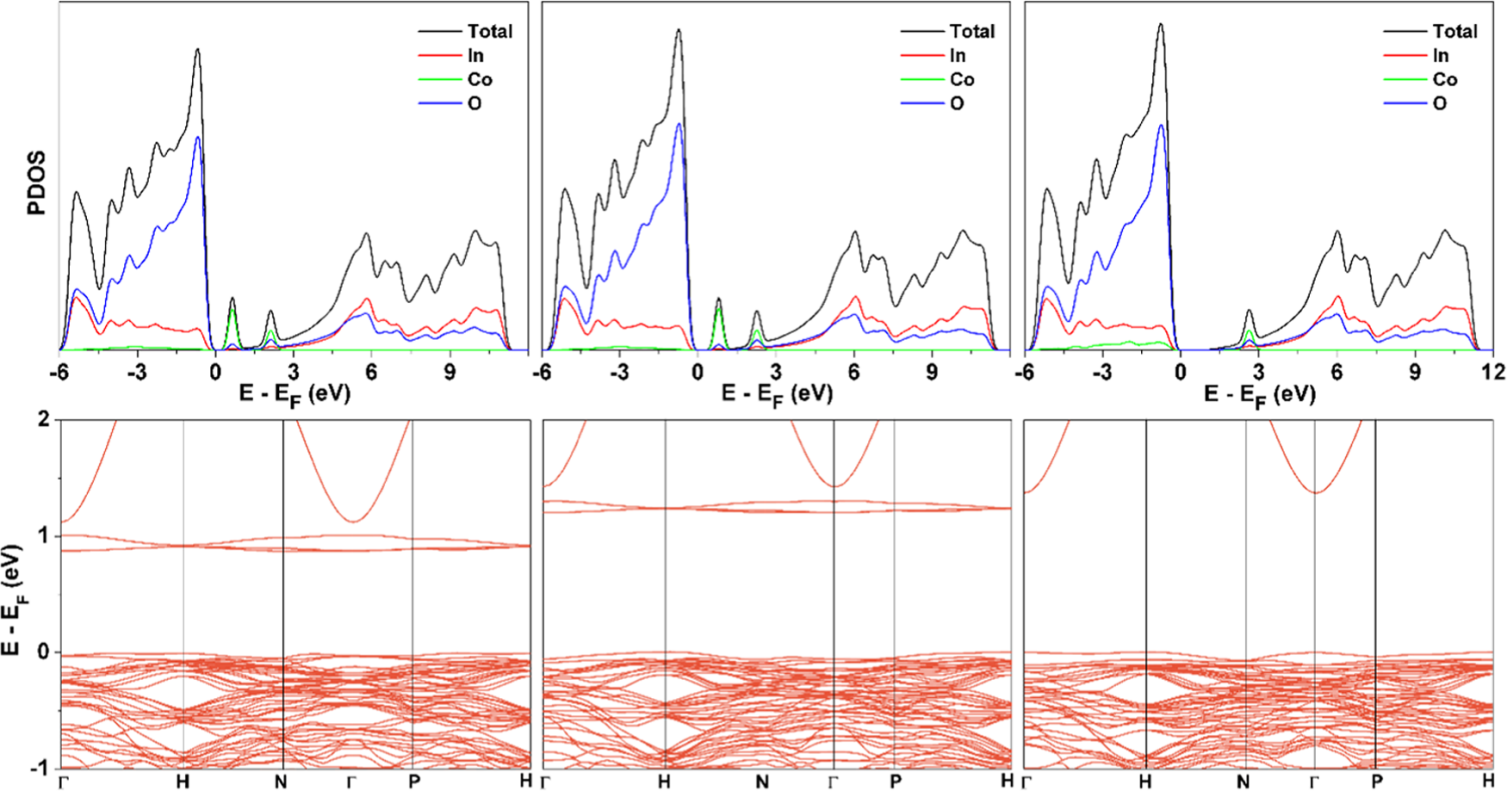
Density of states for Co-doped bcc In_2_O_3_ (3.125 atom %) using PBE (top, left), PBE + *U* (top center for *U*_In_(4d) = 7 and *U*_Co_(3d) = 0 eV), and PBE + *U* (top right for *U*_In_(4d) = 7 and *U*_Co_(3d) = 5 eV). The corresponding band structures are displayed in the bottom panels. All energies are referred to Fermi energy, *E*_F_, defined here as the maximum of the valence band.

Let us now focus on the relevant properties of Co-doped In_2_O_3_ materials. A first important issue concerns the oxidation state of the dopant. To this end, we rely on the calculated Bader nets charges which, in all of the explored situations, is in the 1.30–1.50 range. This suggests that one can assign a +2 formal oxidation state to the dopant. This is not surprising as the charge of the In atom in the ground state electronic structure of In_2_O_3_ is also closer to +2 than to +3 corresponding to a fully ionic system, suggesting a 3d^7^ electron configuration in cobalt (Co) with a possible localized magnetic moment in Co. However, all spin-polarized calculations, whether employing PBE or PBE + *U* with *U*_In_(4d) = 7 and *U*_Co_(3d) = 0 eV or *U*_In_(4d) = 7 and *U*_Co_(3d) = 5 eV, consistently converged to a closed-shell state irrespective of the initial magnetic moment in the Co atom. This observation indicates that the covalent contribution to the bond within this system, evidenced in the DOS in Figure 8, suppresses the spin in the dopant. This outcome is in good agreement with the assignment from the experiment shown in Figure 4.

A second important issue concerns the lattice contraction triggered by Co doping as evidenced by experiment (cf. Figure 3). Table 3 reports the predicted numerical values of the lattice parameter *a*, obtained from energy minimization with respect to both the lattice and fractional coordinates. These results show a significant and gradual variation of the lattice parameter, yielding a lattice contraction that varies almost linearly with increasing Co content from 1.563 up to 9.375 atom % of Co. It partially agrees with the experimental results reported in Figure 3 for atom % of Co ranging from 1.0 to 5.0. The lattice contraction can be easily understood as a response to the presence of atoms with lower atomic radii in the lattice. Note, however, that for Co doping concentrations larger than 5 atom %, the experimental values in Figure 3 show a change in the trend which is not easy to interpret considering exclusively doping-defect effects.

**Table 3:** Dopant Concentration (Atom %) Indicating the Employed Supercell Dimensions, and the Number and Type of Co Atoms (#Co and Either In_1_ or In_2_)^a^

atom %	supercell	#Co	*a*	*E*_f_
0	111	0	10.227	
1.563	211	1 (In_1_)	10.209	2.00
3.125	111	1 (In_1_)	10.198	1.71
3.125	211	2 (In_1_)	10.199	2.02
6.250	111	2 (In_1_)	10.170	1.79
9.375	111	3 (In_1_)	10.126	1.94

a The optimized lattice parameter, *a*, in Å, and dopant energy formation, *E*_f_, in eV, are also reported, which, for each dopant concentration, corresponds to the most stable situation. Note that the same supercell is used for different situations.

The Co doping formation energies for different Co doping situations estimated as in eq 1 are summarized in Table 3, where the most stable situation, among the explored ones, is reported. For the substitution of a singly In atom by one Co atom in the 111 supercell containing 32 In and 48 O atoms, the Co concentration is 1/32 or 3.125 atom %. Here, the only two possible situations involve substituting either one In_1_ or one In_2_ atom; both cases feature positive formation energies but the former leads to a lower *E*_f_ of 1.71 eV. The same concentration can be simulated by the 211 supercell but substituting two In_1_ atoms by Co. The *E*_f_ thus computed is 2.02 eV, which is reminiscent of the situation discussed above for the formation of an O vacancy formation. The 0.3 eV energy difference is also attributed to differences in the degree of completeness of the basis set in the two supercells and, again, provide error bars for the estimate of this property. Substituting two In atoms by two Co atoms in the 111 supercell leads to a Co doping concentration of 6.250 atom % as 2 of the 32 In atoms in the cell are substituted by Co. Here, the possible situations involve substituting either one In_1_ and one In_2_ atom or both two In_1_/In_2_ and, in addition, one needs to consider the possibility of these atoms being near or far of each other. For this Co concentration, the lowest *E*_f_ value is 1.79 eV, and it corresponds to the case in which two In_1_ atoms are close together. Substituting three In atoms by three Co atoms in the 111 supercell implies a Co concentration of 9.375 atom % in Co. Also, in this case, all of the possible combinations were considered, and, in analogy to the previous case, the most stable situation involves three close In_1_ atoms with a slightly larger of 1.94 eV. The situation just described is also found when considering the larger supercells featuring smaller Co concentrations. Overall, the most stable situation involves substituting In_1_ atom and, in cases with more than one substitution, or nearby In_1_ atoms. In conclusion, for the different Co doping situations considered, the computed Co doping formation is roughly 2 eV, see Table 3, and for the range of Co doping explored, the formation energy is quite insensitive to the Co concentration.

One can summarize the preceding discussion by noticing that, for the concentration of the O_vac_ explored, the predicted energy formation is in the 2.5–3.0 eV range, whereas Co doping has a cost of roughly 2 eV per Co atom introduced to the unit cell. Also, the formation of O vacancies has little effect on the lattice parameter, whereas the presence of Co induces a monotonous decrease. However, Figure 3 shows a clear change in the trend of the lattice parameter as a function of the Co concentration in such a way that a higher Co concentration does not induce further changes on the lattice parameter. One could argue that upon doping, the number of O vacancies increases, which would then counteract the effect of the Co atoms. To investigate whether this is a plausible explanation, the energy cost of introducing O vacancies in the Co-doped unit cell has been considered. The results indicate little variation in the O_vac_ formation energy, indicating that this explanation can be ruled out. The fact that experiment and theory coincide for low Co concentration strongly suggests that the computational modeling is sound. Based on this, it is clear that the computational model at Co concentration higher than 5 atom %, even including the presence of oxygen vacancies, does not represent the experimental situation or, alternatively, that the experimental situation at this high Co concentration does not correspond to bulk doping and that the Co atoms are progressively added to surface sites, likely suffering some type of aggregation as previously mentioned. This interpretation is also in agreement with the fact that there is an energy penalty for every substitution of In by Co that increases with the number of added Co atoms, as well as the above-mentioned discussed behavior of the Co/In XPS ratio (cf. Figure 5). New experiments and computational modeling will be carried out to analyze the effect of Co doping across different surfaces, both surface and subsurface doping scenarios, alongside exploring the interactions between undoped and doped surfaces with CO_2_. The effect of the particle size on the electronic properties of stoichiometric and Co-doped In_2_O_3_ samples represents yet an additional aspect that needs also to be considered. This requires handling nanoparticles containing explicitly hundreds of atoms, as done, e.g., for TiO_2_ and ZnO,^55,56^ which is nowadays doable although it requires considerable computational resources.

We finally comment on the effect of Co on the electronic structure of the doped material. For a Co concentration of 3.125 atom %, the PBE + *U* —*U*(In_4d_) = 7 eV and *U*(Co_3d_) = 5 eV—band gap is 1.37 eV with the Co states close to the valence band, as reported in Figure 8. This is almost the same as that in the stoichiometric In_2_O_3_ material (1.38 eV), showing no relevant effect of the cobalt on the band gap. This result agrees well with the present experimental value for stoichiometric In_2_O_3_ also reported in Table 1, which indicates that there is no clear variation of the band gap for the doped material with respect to stoichiometric material. The presence of gap states is also apparent, which is in line with experiment.

## 5 Conclusions

In the context of thermo-, photo-, and dual thermo-photocatalytic applications for the CO_2_ reduction reaction, Co doping is regarded as a promising strategy to tune the In_2_O_3_ efficiency. To better understand this new system, the role of Co in the atomic and electronic structures of stoichiometric and reduced In_2_O_3_ has been studied. A series of experimental results and theoretical calculations within the DFT framework were presented. The analysis of Bader charges shows that this material largely departs from the full ionic picture, with charges on In close to +2 indicating that this is the oxidation state adopted by Co in the doped material, in agreement with the interpretation of XPS experiments. The experimentally observed decrease of the lattice parameter is well reproduced by the calculations although for small dopant concentration only, indicating that at large dopant concentration, the Co atoms are likely to accumulate at or close to the surface of the materials. On the other hand, the lattice parameter is quite insensitive to the concentration of O vacancies, and the formation energy for these vacancies is roughly 2.5–3.0 eV. Similarly, the formation energy of the doped material is quite insensitive to the Co concentration. Our thorough investigation can help to understand the structural, energetic, and electronic effects of these doped materials useful for the subsequent study of the catalytic valorization of CO_2_ using Co-doped In_2_O_3_.
